# Imaging of Status Epilepticus

**DOI:** 10.3390/jcm14092922

**Published:** 2025-04-23

**Authors:** Pilar Bosque Varela, Lukas Machegger, Bernardo Crespo Pimentel, Giorgi Kuchukhidze

**Affiliations:** 1 Department of Neurology, Neurocritical Care and Neurorehabilitation, Christian Doppler University Hospital, Centre for Cognitive Neuroscience, Member of the European Reference Network EpiCARE, Paracelsus Medical University of Salzburg, 5020 Salzburg, Austria; pi.bosque-varela@salk.at (P.B.V.); bernardo.pimentel@gesundheitsverbund.at (B.C.P.); 2 Department of Neuroradiology, Christian Doppler University Hospital, Paracelsus Medical University, 5020 Salzburg, Austria; l.machegger@salk.at; 3 Department of Neurology, Hietzing Hospital and Neurological Center Rosenhügel, 1130 Vienna, Austria; 4 Neuroscience Institute, Christian Doppler University Hospital, Centre for Cognitive Neuroscience, 5020 Salzburg, Austria; 5 Karl Landsteiner Institute for Neurorehabilitation and Space Neurology, 5020 Salzburg, Austria

**Keywords:** status epilepticus, MRI, peri-ictal MRI abnormalities, outcome

## Abstract

MRI plays an increasingly important role in the diagnosis of status epilepticus (SE). Approximately half of patients with SE do not have pre-existing epilepsy, and the cause of de novo SE is frequently unknown. The role of MRI in the identification of causes of SE is invaluable. MRI is often helpful as a diagnostic tool in cases of non-convulsive status epilepticus (NCSE) with ambiguous EEG findings. Thus, MRI is recommended for all patients presenting with de novo SE, patients with known epilepsy with the first episode of SE and NCSE with equivocal EEG. Different peri-ictal MRI (PMA) alterations may be seen during ongoing SE or briefly after its cessation. They commonly present as peri-ictal hyper-perfusion, diffusion restriction and/or FLAIR-hyperintensity affecting specific brain areas such as the cortex, hippocampus, pulvinar of the thalamus, splenium of the corpus callosum, claustrum or cerebellum, frequently in combination, suggesting the existence of a “status epilepticus network”. MRI sequences, which are necessary for detecting PMA, include diffusion-weighted imaging, fluid attenuated inversion recovery, T1-weighted imaging with and without contrast application, as well as perfusion sequences such as arterial spin labeling. Recent research suggests that they may serve as biomarkers for predicting an outcome in SE. Patients with PMA seem to have a higher mortality rate compared to those without PMA. However, there is still a substantial knowledge gap and there are many open questions related to imaging in SE. Further prospective quantitative MRI studies with uniform protocols, timing and follow-up periods are needed to answer these important and clinically relevant questions.

## 1. Introduction

### 1.1. Status Epilepticus

Status epilepticus (SE) is a neurological emergency defined as “a condition resulting either from the failure of the mechanisms responsible for seizure termination or from the initiation of mechanisms, which lead to abnormally prolonged seizures (after time point t1). It is a condition that can have long-term consequences (after time point t2), including neuronal death, neuronal injury, and alteration of neuronal networks, depending on the type and duration of seizures” [1]. SE is a common condition, with an estimated 50,000–60,000 new cases per year in the USA [2]. SE is caused by various conditions, such as acute stroke, encephalitis, cerebral trauma, etc. It may be associated with high mortality and morbidity depending on its etiology, duration, level of consciousness, and semiology [3,4]. The new definition of SE incorporates two time points: t1—the time point when the treatment of SE should start—and t2—the time point after which the ongoing seizure activity may cause neuronal injury and even neuronal death with corresponding long-term consequences [1]. Therefore, it is of decisive importance to diagnose SE timely, identify its causes, and initiate its treatment immediately. It is recommended to perform either computer tomography (CT) with contrast application and CT angiography or magnetic resonance tomography (MRI) with perfusion sequences in order to determine the causes of SE.

### 1.2. Imaging of Status Epilepticus

One of the first reports of multimodal neuroimaging in status epilepticus (SE) was published in 1989 [5]. In this case report, typical findings in different imaging modalities (CT, MRI and SPECT) were described in a 35-year-old woman with longstanding epilepsy and focal motor SE with impaired consciousness. The patient had clonus in the left half of the face, including the eyelids; on EEG, rhythmic activity was registered in the right fronto-centro-temporal area. Single Photon Emission Tomography (SPECT) performed on the 3rd day of SE showed prominent hyper-perfusion in the right central area; on cerebral CT taken on the same day, swelling in the right fronto-central area was observed, and MRI (performed on the 5th day of the ongoing SE) demonstrated blurring of the grey/white matter junction, swelling and leptomeningeal enhancement (after application of Gadolinium) in the right fronto-central area. On the follow-up CT two weeks after cessation of SE, swelling was completely resolved [5].

Despite significant advances in different modalities of brain imaging in recent years, especially in MRI, it has received relatively little attention in the field of SE as opposed to SE semiology, EEG and treatment. The majority of the existing literature on neuroimaging in SE consists of case reports, small case series and most of the studies are of a retrospective nature. MRI, however, is a crucial test in many cases of SE. Approximately 50% of patients develop SE without pre-existing epilepsy, and therefore, the cause of de novo SE is frequently unknown [2]. Acute and subacute structural causes of SE, such as brain trauma, encephalitis, ischemic or hemorrhagic strokes, are well identified by MRI, and therefore, the role of MRI in determining the treatment and the prognosis of SE is invaluable [6].

MRI manifestations of SE are useful as a diagnostic tool in cases of non-convulsive status epilepticus (NCSE), since EEG may be ambiguous and non-specific. MRI is helpful in establishing the diagnosis of SE in more than one-third of patients in addition to those in whom SE was determined by clinical presentation, EEG, laboratory tests and computer tomography [6]. There are frequent examples from everyday clinical practice and from the literature when an MRI alone provides an opportunity for early identification of alterations related to seizure activity as well as underlying structural brain abnormalities [7]. Some even believe that diffusion-weighted imaging (DWI) sequences, for instance, can be more sensitive in detecting epileptogenic areas as compared to post-ictal EEG (EEG sensitivity drops dramatically with the time after cessation of a seizure or a SE) [8].

Thus, MRI is recommended for all patients presenting with de novo SE, patients with known epilepsy with the first episode of SE and in NCSE with equivocal EEG [9]. The MRI sequences, which can reliably detect SE-related abnormalities, include, among others, diffusion-weighted imaging (DWI), fluid attenuated inversion recovery (FLAIR), arterial spin labeling (ASL) and T1-weighted imaging with and without contrast application [10,11,12].

Variable peri-ictal MRI alterations [11,13,14] (PMA) can be seen in patients with an ongoing SE or briefly after its cessation. Diffusion restriction [8,14] and increased signal in FLAIR, which may appear at the same time [15], are the most common PMA. These MRI alterations reflect a mixture of cytotoxic and vasogenic edema; their appearance depends on MRI timing after the onset of SE [13,14]. Another diagnostically important MRI feature of SE is ictal hyper-perfusion [16], which is commonly seen as the first MRI manifestation of SE. It is equally well depicted by contrast-enhanced perfusion sequences and ASL [14,17]. Peri-ictal hyper-perfusion in ASL is seen in 37–100% of patients, depending on patients’ selection (prospective or retrospective series) and timing of the MRI [11,18]. Based on our own data [11] and other studies [18], the best time window for depicting peri-ictal hyper-perfusion is the 24–48 h after the onset of SE. Diffusion restriction and FLAIR-hyperintensity are seen in fewer patients as compared to peri-ictal hyper-perfusion, in 27% and 18%, respectively [11].

**Figure 1:**
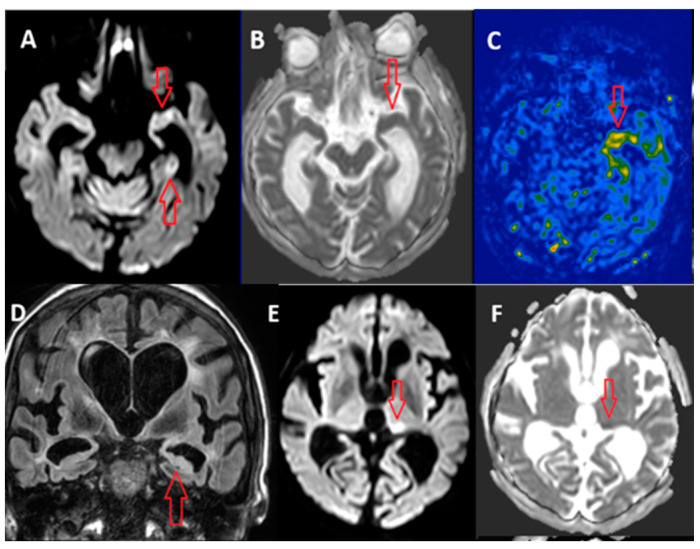
(personal courtesy): Example of PMA in the acute MRI in a patient with NCSE taken in less than 24 h after the onset of SE: diffusion restriction (**A**), normal ADC (**B**), hyper-perfusion in ASL (**C**) and signal increase in FLAIR (**D**) in the left hippocampus (red arrow). Diffusion restriction (**E**) and signal decrease in ADC map (**F**) on the left pulvinar of the thalamus (red arrow).

Alterations related to SE have usually been seen in specific cerebral locations, as demonstrated by the majority of imaging studies, most of which are of a retrospective nature. These areas involve the cerebral cortex, pulvinar of the thalamus, hippocampus, claustrum and cerebellum (Figure 1) [19,20].

In a rare prospective case series of 54 patients with SE, the hippocampus was the most frequently affected brain structure (68.5%), followed by the pulvinar of the thalamus (25.9%), which was involved mainly together with the hippocampus (71.4%); in 24.1%, changes in DWI were registered only in the neocortex [21]. PMA (mainly diffusion restriction) were ipsilateral to the seizure onset zone in the majority of cases (81.5%); in the rest of the bilateral/contralateral cases, propagation patterns were presumed [21]. In a larger study of 106 patients with SE, in total 42.5% had diffusion restriction either only in the neocortex (24.5%) or in both the cortex and ipsilateral pulvinar (17.9%) [22]. Longer duration of SE was associated with the simultaneous involvement of the cortex and pulvinar of the thalamus, favoring the hypothesis of the spreading pattern of seizure activity via cortico-pulvinar connections [22]. Involvement of the pulvinar nucleus of the thalamus in SE may be associated with disturbance of consciousness [21]. The hippocampus is widely known to be involved in seizure propagation, sometimes resulting in hippocampal sclerosis following prolonged seizure activity [23].

Another brain structure in which PMA are frequently observed is the splenium of the corpus callosum. Splenial diffusion restriction and T2-weighted signal are most likely to be associated with ongoing seizure activity and are reversible in most cases [15,24]. Some authors suggest that reversible diffusion restriction in the splenium of the corpus callosum is associated with bi-temporal seizure onset and psychiatric comorbidities in patients with epilepsy [25]. Despite these observations made in a relatively small retrospective case series, it is not entirely clear whether these structures are typically affected in patients with SE or in those with single seizures as well.

The MRI changes related to SE are entirely reversible in the majority of cases [26], however, it is not well known when they appear after the onset of an SE [27]. These changes are variable from patient to patient, as presented in different, for the most part, retrospective case series [15]. In a fraction of patients, mostly in those with refractory SE, PMA persist over days and even weeks, causing permanent damage to the brain in the form of hippocampal atrophy [13,21,26] or focal cortical thinning [15,16].

## 2. Differential Diagnosis of SE in MRI

Vasogenic and cytotoxic cerebral edema may occur in SE depending on its severity, etiology and duration. Intracellular collection of liquid due to failure of an ATP-dependent Na+/K+ membrane pump and intracellular influx of Ca++ causes cytotoxic edema [28]. On MRI, it is depicted as a diffusion restriction on DWI and a signal decrease on ADC. Vasogenic edema, however, contrary to cytotoxic edema, is associated with extracellular water accumulation and damage to the blood-brain barrier. Signal increases in T2-weighted sequences without diffusion restriction and contrast enhancement are typical for vasogenic edema on MRI [28].

Diffusion restriction and cerebral edema in SE should be differentiated from other conditions, such as acute stroke, encephalitis or brain tumor. It becomes rather complicated if these disorders are causes of SE.

### 2.1. Stroke

In patients treated in stroke units, stroke mimics are present in 2–30% of cases, and acute seizures or SE comprise approximately 20% of stroke mimics [29]. The majority of patients in whom stroke mimics are suspected have, in fact, either remote symptomatic epileptic seizures or an NCSE [30]. In children, seizures that present as a stroke mimic are usually associated with acute neurological illness [31].

There are several key points that may help in discriminating SE from stroke if diffusion restriction occurs on MRI: 1. The alterations related to SE do not respect vascular irrigation areas [20]. 2. The signal intensities on DWI and ADC are not as prominent in SE as in acute stroke [32]. Quantification of DWI and ADC signal intensities may be helpful in differentiating acute stroke from SE [32]. 3. In SE, diffusion restriction and FLAIR-hyperintensity may be seen at the same time, whereas in acute stroke, diffusion restriction usually occurs before FLAIR changes are visible. 4. MRI sequences related to cerebral blood perfusion (ASL—arterial spin labeling [7], MRI perfusion with contrast substance, To—time of flight, SWI—susceptibility weighted imaging [33,34]) are of paramount importance for differentiating between ongoing ictal activity (hyper-perfusion) and acute stroke (decreased perfusion) [16]. 5. Importantly, SE-associated diffusion restriction (as well as other MRI changes) usually resolves in a matter of days or weeks, whereas MRI changes due to stroke are persistent.

In a study on 10 patients with an SE, high signal in DWI was less intense than in stroke, and the ADC hypointensity was 13.1% lower than that of the opposite healthy side—that is, one-third compared to the difference in an acute ischemic stroke [35]. All patients had hyper-perfusion in ASL and three of them had additional hyper-perfusion in the ipsilateral pulvinar of the thalamus. Interestingly, SE did not show alterations in medullary venous intensity on SWI, which is frequently observed in patients with an acute ischemic stroke [35]. Another comparative study on patients with SE (N = 26) and acute ischemic stroke (N = 164) proposed cut-offs of signal intensities in DWI and ADC for better differentiating between these two entities [32].

### 2.2. Encephalitis

MRI features of encephalitis and SE may largely overlap, making the differential diagnosis rather challenging. T2-weighted hyperintensities, including FLAIR, diffusion restriction and disruption of a blood-brain barrier, may occur in both SE and encephalitis [36,37]. There are, however, some features that point out more to limbic encephalitis rather than SE-associated MRI changes: bilateral involvement, confinement to limbic structures and more prominent contrast enhancement as opposed to SE. On the other hand, involvement of extra-temporal areas with simultaneous hyper-perfusion would suggest more SE-related MRI alterations. As opposed to MRI features of encephalitis, which usually progress over time, PMA are reversible in a matter of a few days or weeks in most cases [36,37]. Distinguishing between MRI alterations of SE and encephalitis should take into consideration electro-clinical features of individual patients.

### 2.3. Brain Tumors

Up to 70% of patients with brain tumors experience epileptic seizures, including SE [38]. SE-associated edema, presented as a signal increase in T2-weighted images on MRI, may be mistaken for a brain tumor, or conversely, a mass lesion may be misinterpreted as an SE-related MRI change.

MRI changes caused by seizure activity may be also mistaken for tumor progression, and unnecessary treatments may be initiated. As opposed to other features of SE (diffusion restriction, FLAIR-hyperintensity, hyper-perfusion), strong contrast enhancement is frequently observed in tumor patients after experiencing seizures or SE. This could be due to the increased permeability of the blood-brain barrier caused by previous radiation therapy or by tumor neurobiology itself. These observations are based solely on a few small case series and need further systematic investigation [39,40]. Therefore, a series of follow-up MRIs are of vital importance for differentiating SE-associated abnormalities from brain tumors [41].

## 3. Perfusion MRI in SE

Imaging of cerebral perfusion is increasingly recognized as a biomarker of elevated metabolic demand during ongoing seizure activity or as an indicator of post-ictal dysfunction [11]. SE-associated perfusion changes have been described in a number of studies and can be detected with or without a Gadolinium injection.

Conventional T2-weighted perfusion MRI is performed with a contrast substance, Gadolinium.

### 3.1. Arterial Spin Labeling (ASL)

ASL is a non-invasive, easily reproducible alternative to a conventional MRI perfusion with a contrast substance. It uses magnetized water molecules in the blood as an internal contrast [42]. ASL has been widely used for detecting local perfusion changes in patients with epilepsy. Post-ictally, after a habitual seizure, ASL shows hypoperfusion, as observed in a recent prospective study on 21 patients with epilepsy [43]. The majority (71%) demonstrated hypoperfusion 90 min after the seizure, and in 80%, focal hypoperfusion co-localized with the presumed seizure onset zone [43].

In some retrospective studies (small case series), it has been demonstrated that up to 65% of patients with convulsive SE [18] and up to 73% of patients with NCSE [44] focal hyper-perfusion in ASL can be seen. It was observed in the cortex, pulvinar of the thalamus, hippocampus and contralateral cerebellum [18]. In the majority of cases, the ASL hyper-perfusion resolves in approximately a week; in some cases, however, ALS hyper-perfusion persists up to four weeks, exceeding ictal activity on EEG [44].

ASL is characterized by the highest sensitivity in detecting SE-related changes as opposed to other MRI sequences such as DWI, FLAIR and dynamic susceptibility contrast (DSC). In a study comparing MRI alterations in SE and self-limiting seizures, ASL acted as the most sensitive method for differentiating between MRI features of the two conditions [45]. Altered perfusion was seen in 90.2% of SE patients as compared to 41.3% of patients with self-limiting seizures. The majority of patients with a SE showed peri-ictal hyper-perfusion (91%); whereas in those with self-limiting seizures, almost half of the patients demonstrated post-ictal hypoperfusion [45]. However, the specificity of ASL alterations in general for differentiating SE from single seizures was modest (58.7%) [45]. The specificity of changes in ASL in detecting SE increases dramatically if they are observed in the thalamus (especially in its pulvinar nucleus)—61.2–100% [45,46]. Simultaneous hyper-perfusion in the neocortex, nucleus pulvinar of the thalamus and hippocampus strongly suggests PMA [47].

ASL also demonstrated an excellent sensitivity for predicting refractoriness of an SE (89.5%) and its poor outcome (100%) as opposed to other MRI sequences, an EEG and clinical outcome scores. However, here the specificity of ASL was rather low: 9.4% (SE refractoriness) and 15.6% (poor outcome) [45].

### 3.2. Susceptibility Weighted Imaging (SWI)

Cerebral perfusion can be assessed also by susceptibility weighted imaging (SWI), an MRI sequence, which is routinely used for identifying intracranial bleeding, cerebral venous thrombosis, vascular malformations and cerebral calcifications. In acute stroke and migraine, cortical veins appear dark and prominent on SWI due to high levels of deoxyhemoglobin with paramagnetic properties, suggesting hypoperfusion in the affected areas [48,49,50]. In some retrospective studies, SWI has been used for assessment of cortical veins in patients with acute seizures [33,34]. In a study on 12 patients with SE (6 with convulsive SE and 6 with NCSE), all patients demonstrated local pseudo-narrowing of cortical veins on SWI and corresponding hyper-perfusion on conventional, gadolinium-enhanced MRI-perfusion [33]. In half of patients, cortical diffusion restriction in hyperemic areas was observed [33]. The same group of authors presented other retrospective series of 26 patients with either convulsive SE or NCSE without clear timing of SE onset in most cases [34]. The majority of patients (23/26, 88%) presented with either a global or focal pattern of hyper-oxygenation on SWI sequence with a corresponding increase in regional cerebral blood flow, suggesting an ictal increase in cerebral metabolism. In a small proportion of patients (3/26, 12%), SWI demonstrated focal hypo-oxygenation along with a local decrease in cerebral perfusion as assessed by conventional MRI perfusion with Gadolinium, suggesting post-ictal hypometabolism and dysfunction [34].

## 4. Irreversible Changes (Brain Atrophy/Hippocampal Sclerosis) on MRI Due to SE

Long-term consequences of prolonged SE include, among others, cortical laminar necrosis [51,52] and hippocampal sclerosis [52,53,54,55]. These alterations usually develop following PMA in the form of diffusion restriction and FLAIR-hyperintensity (cytotoxic edema), as demonstrated by experimental and some clinical studies. PMA persisting for days to weeks eventually results in a brain tissue volume loss [51,56,57,58,59]. In case reports or small case series, focal brain atrophy was seen on MRI after three to four weeks following the SE, where on initial MRI, focal diffusion restriction, edema and hyper-perfusion were observed [60]. Hippocampal sclerosis may develop as a result of a febrile SE as shown in a longitudinal MRI study on 11 children with a febrile SE. On average, 9 months following an SE, 5/11 children developed hippocampal sclerosis [55]. The risk factors for developing post-SE hippocampal damage or other permanent brain alterations in an individual patient are unknown: particular types of SE-associated MRI abnormalities, types of SE and duration of SE, its etiology or certain EEG patterns. In a recent prospective MRI study on 353 patients with an SE, we performed two follow-up MRIs when PMA were present on a previous MRI [52]. On an acute MRI, PMA were seen in 44% of patients (156/353); in over half of patients, PMA were reversible after one week. In those with persisting PMA, another follow-up MRI was performed four weeks after the initial acute MRI, showing signs of focal brain atrophy, mostly in the hippocampus. In total, volumetric analysis demonstrated decreased brain volume in 85% of patients with a median volume loss of 16%. Decrease in cerebral volume correlated with the duration of SE and the length of required hospitalization [52].

## 5. Prognostication of SE Outcome Based on Its MRI Manifestations

SE is associated with high mortality and morbidity. Over the years, predicting outcomes in SE has been a primary focus of research in the field and a subject of extensive discussion among experts. The ability to forecast outcomes during an acute phase of an SE holds the potential to guide the initiation of targeted treatments, which is especially important for NCSE, where treatment approaches are sometimes controversial [61]. Although predicting mortality is vital, the need for outcome tools that also assess favorable functional long-term outcomes is crucial for avoiding potentially harmful treatment regimens in SE. Furthermore, understanding the likely functional outcome in patients with refractory SE is essential for determining the appropriate duration of treatment and considering alternative therapeutic approaches [62]. Currently, specific validated outcome markers remain scarce, primarily due to the semiological heterogeneity of SE. However, there is a substantial amount of evidence for predicting short-term mortality [63]. Several factors have been associated with higher mortality rates, including etiology [64,65], age [66,67], duration of SE [66], level of consciousness [3,4], EEG features—the presence of periodic epileptiform discharges is linked to a worse outcome [68]. To assess the outcome of patients with SE, four major prognostic scores have been developed and validated: EMSE [3], STESS [69], mSTESS [70] and ENDIT [71], relying on demographic data, clinical, and electroencephalographic features, among others. Their main aim of these scores is predicting adverse outcomes, in particular short-term mortality (strong and weak points of these clinical outcome scores are detailed in Table 1). Moreover, different serum and cerebrospinal fluid (CSF) have been identified in SE [72,73,74]. Specific proteins are released into CSF and serum due to ongoing neuronal-axonal damage or inflammation. However, a significant limitation of these prognostic scores is their inability to adequately assess the severity of brain injury or functional outcomes in surviving patients. Moreover, they have not demonstrated sufficient accuracy in predicting refractoriness to treatment [75]. Consequently, identifying patients at risk of developing refractory SE and, subsequently, brain injury remains a significant knowledge gap in the field.

In recent years, MRI has been primarily employed for diagnosing the underlying causes of SE. However, PMA may also serve as a potential prognostic biomarker. Bonduelle et al. conducted a retrospective study where they compared in-hospital mortality in patients with SE and PMA as opposed to those with SE and without PMA [10]. PMA were observed in 26% of 307 patients with SE [10]. In 15% of this population, an in-hospital death occurred, and it was significantly higher in patients with PMA vs. those without PMA. There were, however, other independent factors that were closely associated with an increased mortality along with PMA. These were duration of SE, older age, acute lesions on MRI and potentially fatal comorbidity [10]. In addition, the presence of PMA was associated with a higher rate of refractory SE and, in surviving patients, with developing epilepsy after the episode of de novo SE [10].

In another retrospective study on 101 patients with SE, ictal MRI abnormalities were linked with neurological deterioration at discharge, irrespective of the cause of SE [76]. They were associated with a longer duration of SE and a significantly higher mortality rate in patients with non-acute etiologies [76].

These findings suggest that PMA may serve as a potentially useful biomarker for predicting the short-term outcome of SE. However, it remains open whether PMA could also forecast a long-term outcome; this field is under investigation and necessitates further research.

## 6. Conclusions

In summary, although the diagnosis of an SE relies on a combination of electro-clinical findings [1], neuroimaging, especially an MRI, is a key component in the diagnostic work-up and management of SE; in many cases, it is crucial in terms of the diagnostic value and immediate intervention. Neuroimaging assists in better understanding the pathophysiology of SE and in assessing its prognosis. Recent research suggests that MRI could also serve as a biomarker for predicting outcomes in SE. Patients with PMA seem to have a higher mortality rate compared to those without PMA [10]. However, there is still a substantial knowledge gap, and there are many open questions related to imaging in SE. Further prospective quantitative MRI studies with uniform protocols, timing and follow-up periods are needed to answer these important and clinically relevant questions.

## Figures and Tables

**Table 1:** Strong and weak points of status epilepticus outcome scores.

Outcome Score	Strong Points	Weak Points
EMSE	Includes age, etiology, comorbidity and EEG. Explains individual mortality in 90% of cases. Superior to STESS-3 and STESS-4. Provide a wide quantitative measure for assessing outcome Validated in different cohorts	Does not include level of consciousness and duration of SE. Time consuming Might require specific training for accurate application Underperforms in NCSE patients
STESS	Incorporates level of consciousness, worst seizure type, age and history of seizures Easy to calculate and apply in clinical practice. Focuses on age and SE duration Correctly identifies surviving patients Validated in different cohorts	May oversimplify complex clinical situations by applying limited clinical variables Has a ceiling effect for patients older than 65 years. Low predictive value for unfavorable outcome
mSTESS	Incorporates modified Rankin score into STESS Increases age cutoff from 65 to 70 years	May miss important clinical nuances due to its simplified approach
END-IT	Includes NCSE, diazepam treatment, imaging, tracheal intubation. Has high discriminative power in predicting in-hospital mortality	Underperforms in NCSE patients in the NICU environment

## Abbreviations

MRI	Magnetic resonance imaging
SE	Status epilepticus
NCSE	Non-convulsive status epilepticus
PMA	Peri-ictal MRI abnormalities
CT	Computer tomography
SPECT	Single photon computer tomography
EEG	Electroencephalography
DWI	Diffusion weighted imaging
FLAIR	Fluid attenuated inversion recovery
ASL	Arterial spin labeling
ADC	Apparent diffusion coefficient
ATP	Adenosine triphosphate
ToF	Time of flight
SWI	Susceptibility weighted imaging
